# Epidemic Nephritis

**Published:** 1918-09

**Authors:** 


### ABSTRACTS

**Epidemic Nephritis.** Langdon Brown, Practitioner, Feb., mentions a series of 1062 cases in the English Army in June, 1915, the epidemic having been in progress since Feb. In 1916, even more cases occurred. 166 cases personally observed, showed early dyspnoea in 76%, oedema in 97%, 2 deaths occurring. In 102 cases, the urine was of normal amount but contained blood, epithelial, granular and hyaline casts. Oedema was noted in 18%. Caffeine compounds were considered inadvisable and the principal treatment was rest, restricted diet and hot air baths. No microorganism could be isolated.

Shaw, Dunn and McNee, Brit. Med. Jour., Dec. 8, 1917, state that nephritis has been particularly prevalent in the British Army, rare among Indian troops, in the civil population, and in men not actually at the front, more frequent in enlisted men than officers. Season, locality and bad weather seemed to have no influence. The frequence and precocity of dyspnoea was striking, being noted in 34 of 51 cases. Urinary examinations as found by Brown, bacteriologic examination negative also. Obstruction of the glomerular vessels by cells with almond-like or flattened nuclei and showing mitosis was noted, while the convoluted tubules were in a state of desquamation. The connective tissue of the medullary portion of the pyramids was in a state of hyaline swelling. In the lungs, were noted: acute emphysema, oedema involving the interstitial tissue and the mucosa of the terminal bronchioles and infundibula. There were some punctiform haemorrhages of the subpleural capillaries and thromboses of the alveolar capillaries. Minute haemorrhages of the white matter of the brain with a necrotic center, these lesions and those of the lungs resembling the results of phosgene gas. Relief occurred promptly on rest in hospital, or even promptly on removal from the front. It is implied that the lesions may be due to slight poisoning with phosgene.

Thomas Oliver, Brit. Med. Jour., Dec. 8, 1917, in the main corroborates the preceding but gives the mortality at 3-4: 1000, notes a persistent albuminuria in many cases over 30, and a urinary elimination of lead amounting to more than 1 m.g. a day, in 43%, Powell White, Loeper and Verpy having also found lead poisoning.

